# Ultrasensitive Label-Free Detection of Unamplified Multidrug-Resistance Bacteria Genes with a Bimodal Waveguide Interferometric Biosensor

**DOI:** 10.3390/diagnostics10100845

**Published:** 2020-10-19

**Authors:** Jesús Maldonado, Ana Belén González-Guerrero, Adrián Fernández-Gavela, Juan José González-López, Laura M. Lechuga

**Affiliations:** 1 Nanobiosensors and Bioanalytical Applications Group, Catalan Institute of Nanoscience and Nanotechnology (ICN2), CSIC, BIST and CIBER-BBN, Campus UAB, Bellaterra, 08193 Barcelona, Spain; jesusmalvaz@gmail.com (J.M.); gonzalez.anabelen@gmail.com (A.B.G.-G.); adrigavela@gmail.com (A.F.-G.); 2 Department of Clinical Microbiology, Hospital Univeritari Vall d’Hebron (HUVH), Vall d’Hebron Institut de Recerca (VHIR), Universitat Autònoma de Barcelona, 08035 Barcelona, Spain; jjgonzal@vhebron.net

**Keywords:** nanophotonic biosensor, multidrug-resistance, *Escherichia coli*, bimodal waveguide interferometer, *NDM*, *CTX-M*

## Abstract

Infections by multidrug-resistant bacteria are becoming a major healthcare emergence with millions of reported cases every year and an increasing incidence of deaths. An advanced diagnostic platform able to directly detect and identify antimicrobial resistance in a faster way than conventional techniques could help in the adoption of early and accurate therapeutic interventions, limiting the actual negative impact on patient outcomes. With this objective, we have developed a new biosensor methodology using an ultrasensitive nanophotonic bimodal waveguide interferometer (BiMW), which allows a rapid and direct detection, without amplification, of two prevalent and clinically relevant Gram-negative antimicrobial resistance encoding sequences: the extended-spectrum betalactamase-encoding gene *blaCTX-M-15* and the carbapenemase-encoding gene *blaNDM-5* We demonstrate the extreme sensitivity and specificity of our biosensor methodology for the detection of both gene sequences. Our results show that the BiMW biosensor can be employed as an ultrasensitive (attomolar level) and specific diagnostic tool for rapidly (less than 30 min) identifying drug resistance. The BiMW nanobiosensor holds great promise as a powerful tool for the control and management of healthcare-associated infections by multidrug-resistant bacteria.

## 1. Introduction

Hospital-acquired or healthcare-associated infections (HAIs) refer to an infection that develops at least 48 h after the patient is admitted to a hospital [1]. These infections are caused during a prolonged stay at the hospital and are a major risk factor for serious health issues leading even to death [2]. In Europe, 3–10% of all hospitalizations in acute care hospitals (ACH) and long-term care facilities (LTCF) result in nosocomial infections, with the highest rates in surgical (7%) and intensive care units (19%) [3]. Consequently, it is estimated that a total of 8.9 million HAIs occur each year in European hospitals and LTCF. The most frequently reported microorganisms causing HAIs in Europe during 2016–2017 were Gram-negative bacteria, with *Enterobacterales* (38%) as the most prevalent, of which *Escherichia coli* (16%) and *Klebsiella pneumoniae* (10%) were the most frequently found, followed by other non-*Enterobacterales* Gram-negative bacteria (14%), with *Pseudomonas aeruginosa* (8%) and *Acinetobacter spp* (3%) as the most commonly isolated species [4].

Since their introduction in clinical practice, antibacterial drugs have revolutionized our ability to control infections by dramatically reducing patient morbidity and mortality. However, in recent decades, the massive use of antibiotics has promoted strong selective pressure on bacteria, contributing to the emergence and spread of organisms harboring antibiotic resistance mechanisms [5,6]. Multidrug resistance is now commonly found amongst bacterial pathogens causing HAIs such as pneumonia, surgical site infections, urinary tract infections, and bloodstream infections, and the increase in infections by Gram-negative bacteria resistant to first-line antibiotic treatments such as cephalosporins, carbapenems, fluoroquinolones, and aminoglycosides is especially alarming [7]. According to the data provided by the European Centre for Disease Prevention and Control (ECDC), the rate of resistance to third generation cephalosporins for *E. coli* and *K. pneumoniae* producing HAIs in Europe (from 2016 to 2017) was 21% and 51%, respectively. For *P. aeruginosa* and *A. baumannii*, the rate of resistance to carbapenems was 29% and 72%, respectively. This situation represents an important threat to public health, as these four species account for 72% of the Gram-negative bacteria producing HAIs, and scarce therapeutic options remains available to combat them [2].

One third of HAI, both in ACH and LTCF, are caused by multidrug-resistant (MDR) bacteria. These infections are associated with suboptimal patient outcomes including increased hospital stay, the requirement of advanced medical interventions, the risk of developing sepsis, higher disability and mortality, and increased healthcare costs, which are estimated to be close to $ 9 billion worldwide [5]. Rapid microbiological diagnosis of HAIs produced by MDR bacteria is needed to quickly implement both optimal antibiotic treatment and management strategies, aiming to limit the negative impact on the patient’s outcome. However, routine aetiologic diagnosis is still mainly performed by culture-based methods followed by bacterial identification and antibiotic susceptibility testing techniques, which, although well-established and reliable, have their own limitations. They are time consuming (48–72 h) and expensive and require specific techniques and high expertise. 

New technological approaches are currently being pursued with the aim to develop point-of care devices capable of rapid and cost-effective tests to assist in the diagnosis and management of infectious diseases, which are particularly relevant for critically ill patients. Strategies based on nucleic acid testing, such as those using polymerase chain reaction (PCR) and sequencing, have shown to be highly sensitive tests for bacterial identification [8,9,10,11]. However, PCR techniques are still too expensive, their operation can be complex, requiring trained personal, and they are susceptible to false positive results due to accidental contamination by amplified products.

The detection of unamplified genomic DNA directly from samples, bypassing the standard PCR amplification, with high sensitivity and specificity and in a fast and cost-effective way is a challenge [12]. In conventional techniques such as PCR or microarrays, pre-analytical steps are normally employed prior to genomic DNA detection, such as DNA fragmentation, extraction, and denaturation due to these genes being inside of bacteria [13]. 

Some biosensors have been employed for the direct detection of genomic DNA without previous PCR amplification, such as plasmonic biosensor [14,15,16], piezoelectric [17,18], and optical-fiber-based biosensors [19]. However, the main limitation of those biosensors is their limited sensitivity, which makes necessary the use of secondary amplification steps, such as the use of gold nanoparticles.

Another alternative for bacterial detection is the use of volatile organic compounds (VOCs) [20]. Several analytical platforms have been employed to detect these VOCs related with bacteria detection, such as electronic noses [21], gas chromatography-mass spectroscopy (GC-MS) [22,23], and secondary electrospray ionization-mass spectroscopy [24]. The major limitation of using such VOCs detection technology is the high costs of the instrumentations.

Our approach offers a highly sensitive nanophotonic biosensor for the detection of genomic DNA without any previous amplification, using a bimodal waveguide interferometer device (BiMW). The BiMW biosensor has been widely developed by our group [25,26] and is based on the evanescent field principle, monitoring local changes in the refractive index at the sensor surface. The BiMW sensor consists of a rib single-mode input waveguide followed by a thicker waveguide where two modes (fundamental and first order light modes of the same polarization) travel to the end of the device [27]. By opening a window in the bimodal section, the waveguide is exposed to the external medium, where the bioreceptors are immobilized. When a biomolecular event occurs, the two light modes are affected through their evanescent tails, which induces a phase variation in the interferometric output. This interferometric technology has been already employed for other challenging clinical applications, such as for early cancer diagnosis by analyzing microRNA in urine to stratify bladder cancer patients [28] and for the analysis of mRNA alternative splicing isoforms [29], among others [30]. 

To demonstrate that the BiMW biosensor can be employed as a specific tool for rapidly identify MDR bacteria, we targeted two prevalent and clinically relevant Gram-negative antimicrobial resistance markers: the extended-spectrum betalactamase-encoding gene *blaCTX-M-15* and the carbapenemase-encoding gene *blaNDM-5* [31,32]. We have implemented a methodology using minimal pre-analytical steps such as DNA extraction, fragmentation, and denaturation, allowing the gene DNA detection with the BiMW biosensor. The high sensitivity of the BiMW nanobiosensor allows the rapid detection (≤ 40 min, including sample pretreatment) of genomic DNA (< 1000 mer) at a concentration in the range of 20–30 aM, employing a low amount of DNA sample and without the need for any DNA amplification step. We have demonstrated the capability and applicability of the BiMW biosensor for the detection of multidrug-resistance markers in *E. coli*, without any amplification. This can represent a valuable methodology to be used for HAIs diagnosis and implementing early and accurate therapeutic interventions.

## 2. Materials and Methods

### 2.1. Chemical Reagents

Solvents for the sensor cleaning process, namely dry toluene (> 99.5%), acetone (99.5%), ethanol (99.5%), hydrochloric acid (HCl, 35–38%), and methanol (MeOH, 99.9%), were supplied by Panreac (Barcelona, Spain). Silane-PEG-COOH (600 Da) was purchased from Nanocs (Boston, MA, USA). 1-ethyl-3(3-dimethylaminopropyl) carbodiimide hydrochloride (EDC), N-hydroxysuccinimide (NHS), the components for phosphate buffer saline (PBS; 10 mM phosphate, 2.9 mM KCl, 137 mM NaCl, pH 7.4), 0.5 M NaCl/Tris-EDTA buffer, and 1 mM MgCl_2_ were purchased from Sigma-Aldrich (Madrid, Spain). Polydimethylsiloxane (PDMS) and SU-8 2025 polymers for flow cell fabrication were purchased from Sylgard^®^ (Midland, MI, USA) and Microchem (Newton, MA, USA), respectively.

### 2.2. BiMW Device and Experimental Set-Up

**Figure 1:**
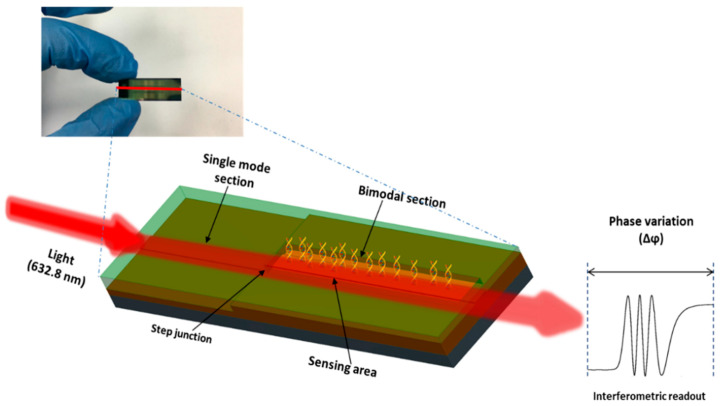
Picture and scheme of a BiMW chip containing 20 sensors, and scheme of the expected interferometric readout.

The working principle of the BiMW interferometric sensor has been widely explained in our previous works [26,27]. In brief, the sensor consists of a straight waveguide supporting two monochromatic light modes of the same polarization in the visible range. The sensor operates by the interference of the fundamental and first order light modes, supporting in a single mode and a bimodal region of the waveguide. The waveguide of the BiMW has a structure of Si/SiO_2_/Si_3_N_4_/SiO_2_ with a width of 2.5–3 µm, a rib height of 2–3 nm, and a length of 3 cm; the thickness of the core for the single-mode and bimodal sections is 150 nm and 340 nm, respectively. A sensing window (15,000 × 50 µm) is defined in the bimodal section of the BiMW. The BiMW devices are fabricated at wafer level in clean room facilities using standard microelectronics technologies. Each BiMW sensor chip has a dimension of 10 × 30 mm and contains 20 independent and identical sensors (Figure 1).

For the evaluation of the BiMW sensors, a bench-top set-up was employed. A He/Ne laser (λ = 632.8 nm) was used as the light source (using TE polarization). The light was end-coupled into the waveguide facet using a 40 × microscope objective. The interferometric bimodal pattern at the output of the bimodal waveguide was captured by a two-sectional Si photodiode, which was placed close enough to the sensor chip output edge for a minimum light dispersion. Each section of the photodiode was connected to a current amplifier in order to amplify the currents. These values were used to calculate the parameter S (signal), according to the Equation (1):

(1) $$S\left(\%\right)=\frac{I_{up}-I_{down}}{I_{up}+I_{down}}\;\alpha \;cos\left[\Delta \phi \left(t\right)\right]$$

where *I_up_* and *I_down_* are the currents measured by the upper and lower sections, respectively, of a two-section photodiode. Δ*ϕ* is the phase variation, which is proportional to the signal *S.* The intrinsic sensitivity of the BiMW sensor to temperature fluctuations was compensated by incorporating a Peltier element behind the sensor chip and a temperature controller, providing temperature stabilization with an accuracy of 0.01 °C. Data acquisition and analysis were performed using home-made LabVIEW (National Instruments, Northampton, MA, USA) software. 

### 2.3. Microfluidic System

A microfluidic cell and a flow delivery system were implemented for the evaluation. For that, a SU-8 2025 master mold was manufactured over silicon wafer using a standard photolithography protocol in clean room facilities, which was replicated in polydimethylsiloxane (PDMS) polymer for microfluidic cell fabrication. Elastomer and curing agent were mixed in a 10:1 ratio, and air bubbles originating from the mixing were removed by a vacuum pump in a desiccator. The PDMS was cured for 1 h at 75 °C on a hotplate to ensure polymer cross-linking. The PDMS microfluidic cell has eight channels, with a separation between them of 250 μm (center to center), with an independent inlet and outlet for each channel. Each microchannel has a length of 18 mm, a height of 50 μm, and a width of 150 μm, which covers the entire sensing window of the BiMW. The microfluidic cell was connected to a syringe pump and an injection valve for fluid delivery. The flow rate was modified according to the bioassay requirements, at a range from 5 to 30 μL min^−1^.

### 2.4. BiMW Surface Cleaning, Activation, and Biofunctionalization

The BiMW sensor surface was deeply cleaned prior to use in order to remove any contamination that can affect the subsequent silanization procedure. The BiMW sensor chips were cleaned with acetone, ethanol, and water, followed by sonication in methanol/hydrochloric acid 1:1 for 10 min, rinsing with water, and drying with a stream of nitrogen. Then, a layer of active hydroxyl group was generated by oxidation using an UV/O_3_ cleaner (BioForce Nanosciences, Salt Lake City, UT, USA) for 1 h, followed by the immersion of the sensor chip in a 10% HNO_3_ solution at 75 °C for 25 min. Clean and hydroxyl-activated sensor chips were immediately incubated with a silane-PEG-COOH solution of 25 mg mL^−1^ ethanol/water 95:5 (*v*/*v*) for 2 h at 4 °C; then, they were rinsed with ethanol and water, and dried with a nitrogen stream. The curing step was performed by placing the sensor chips into a bottle glass in an autoclave for 90 min at 120 °C and a pressure of 1.5 bars. Finally, the immobilization of the DNA probes was carried out ex situ. First, the carboxyl groups on the sensor surface were activated with a solution of 0.4 M EDC/0.1 M sulfo-NHS in MES buffer for 3 h. The amino-modified DNA probe (20 μM prepared in 10 mM PBS containing 1 mM MgCl_2_) was incubated over the sensor surface overnight at room temperature.

### 2.5. Probes Design

Capture probes were designed by the free software OligoArchitectTM. This in-silico evaluation can be applied to select highly performing probes for biosensing applications. In this study, the *blaCTX-M-15* and *blaNDM-5* genes were the DNA target. All the probes were modified with the amine terminal group (5′-amine-probe) in order to react with the carboxyl group introduced on the surface of the sensor chip with the silane-PEG-COOH. Finally, synthetic DNA sequences were used as positive and negative controls. HPLC-purified DNA material was purchased from Ibian Technology, S.L. (Zaragoza, Spain). Detailed information about all the DNA sequences (capture probes and synthetic targets) employed in this study is provided in Table 1. 

### 2.6. Bacteria Culture, DNA Extraction, and PCR Validation

The following bacterial isolates were used: *E. coli* 85BA carrying *blaCTX-M-15*, and *E. coli* 1195rut carrying *blaNDM-5*. Additionally, *P. aeruginosa* and *E. coli*, both negative for *blaCTX-M-15* and *blaNDM-5* were used as negative control. All bacteria were streaked onto Luria broth (LB) and incubated overnight at 37 °C. Purification of bacteria was carried out by centrifugation at 3000 rpm for 15 min at 4 °C. Bacteria were then suspended in PBST. For bacterial DNA extraction, a DNA extraction kit (QIAamp genomic DNA kit, Hilden, Germany) was used according to the manufacturer’s instructions. The extracted DNA samples were analysed by gel electrophoresis, and the concentration of the total bacterial DNA was quantified using a nanospectrophotometer at 260 nm (Nanophotometer™ from Implen, Munich, Germany). The concentrations of the 10-fold diluted extracted DNA from bacteria with the genes were 12.5 pM for the *blaCTX-M-15* gene sample, and 20.91 pM for the *blaNDM-5* gene sample. Regarding negative controls, the DNA concentration from the extraction step was 0.12 nM for *E. coli*, and 71.2 pM for *P. aeruginosa*, respectively. These DNA samples were extracted from a bacteria concentration around 1 × 10^7^ CFU mL^−1^. To validate the presence of the *blaCTX-M-15* and *blaNDM-5* genes, a PCR test of the samples was carried out as previously described [33,34,35,36], and evaluated in agarose electrophoretic gel from amplification with PCR.

### 2.7. BiMW Assay Development

Prior to the BiMW biosensor analysis, all bacteria samples were fragmented by dipping an immersion probe directly in the sample for 15 s at 120 KHz, followed by vortexing for 1 min at 3000 rpm. All double-strand-DNA-containing samples were then heated to 95 °C for 5 min to separate the two DNA strands. Strands re-hybridization was prevented by cooling the samples on ice for 1 min before their introduction into the BiMW biosensor. During all measurements, 0.5 M NaCl/TE (hybridization buffer) was flowed over the sensor surface at a constant flow rate until a stable baseline was reached. The detection of genomic DNA targets was performed by injection of 150 µL of the sample over the surface of the BiMW biosensor at 5 µL min^−1^ rate. In order to reuse the bioreceptor DNA layer after each measurement, an injection of HCl (100 mM) was employed to disrupt DNA-DNA hybridization at 20 µL min^−1^ for 30 s. Calibration curves were obtained by triplicate measurements of different concentrations of genomic DNA. The theoretical limit of detection (LOD) was determined from a linear regression fitting. The data were analyzed using Origin pro 8.0 software (Originlab, Northampton, MA, USA).

## 3. Results and Discussion

### 3.1. Bioassay Strategy and Evaluation with Synthetic DNA Target Sequences

**Figure 2:**
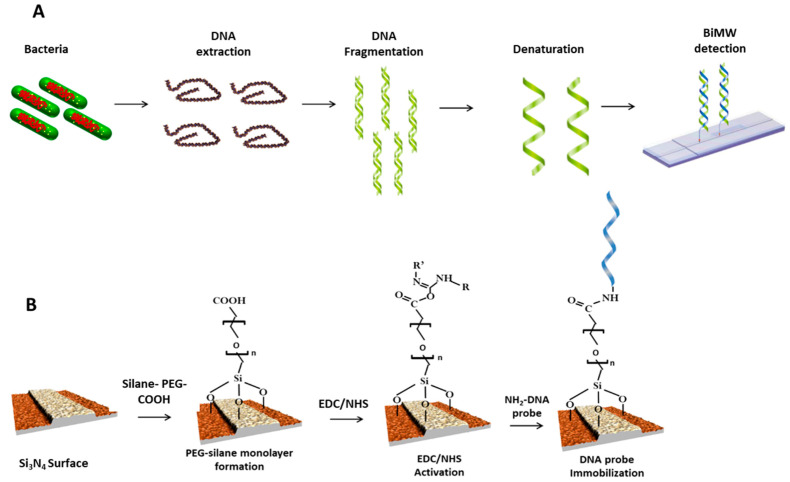
(**A**) Scheme of the biosensor strategy followed for the genes detection. This included DNA extraction, fragmentation, and denaturation to obtain single-stranded fragments, which were then analyzed directly with the BiMW biosensor. (**B**) Biofunctionalisation of the BiMW sensor surface with amine-modified capture probes.

Figure 2A shows the detection strategy proposed for the BiMW biosensor. Following the bacterial lysis for total DNA extraction from the specimen, a genomic DNA fragmentation was done obtaining random DNA fragments between 100 and 1000 bp, which may allow efficient hybridization with the immobilized probe. DNA denaturation of the double-stranded DNA was done using a thermal treatment. Besides, the in silico approach by OligoArchitectTM was used to design the most suitable probes sequences (18–20 mer), which target the most conserved region (1436–2311), in order to assure a high degree of specificity within the corresponding genes. Finally, the genomic DNA detection was performed by hybridization with the specific probes for each gene, previously immobilized on the BiMW sensor surface.

For a proper oligonucleotide immobilization, the silicon nitride (Si_3_N_4_) surface of the BiMW sensor was chemically modified with silane-PEG-COOH in order to generate a homogenous monolayer with anti-fouling properties and exposing reactive carboxyl groups [37]. For the activation of the silane-PEG-COOH monolayer, we employed the well-known EDC/NHS surface chemistry. Figure 2B shows a scheme of the biofuctionalization process. 

**Figure 3:**
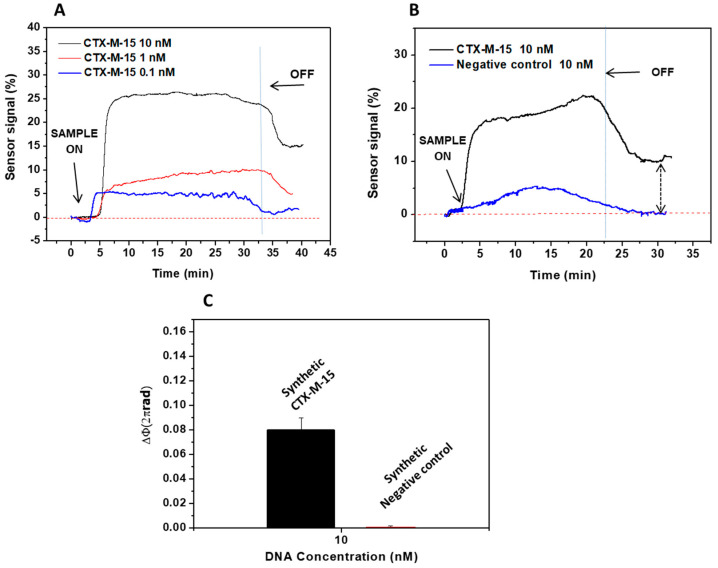
Evaluation of synthetic DNA target. (**A**) Real-time interferometric signal obtained for the hybridization of the synthetic target (88 mer) to *blaCTX-M-15* probe; (**B**) Real-time interferometric signals obtained for the binding of 10 nM of target (black line) and negative control (blue line) to the *blaCTX-M-15*; (**C**) Comparison of the signal of synthetic *blaCTX-M-15* DNA target (10 nM) versus synthetic control target (10 nM). All measurements were done in triplicate, and the data are shown as means + SD.

To demonstrate the capability of the BiMW biosensor for genomic DNA detection, we first evaluated the detection of a 88 mer synthetic DNA target for *blaCTX-M-15* gene using the capture probe (*CTX-M-15* F probe, see Table 1). The probe was previously immobilized onto the BiMW sensor surface, and the corresponding hybridization was monitored at different target concentrations (0.1, 1, and 10 nM) (see Figure 3A). The specificity was also assessed by monitoring the biosensor response after injection of a synthetic negative control at 10 nM concentration. As can be observed in Figure 3B, the negative control sequence recovers the baseline after the running buffer passes over the biosensor surface, while the *blaCTX-M-15* synthetic DNA target produces a clearly noticeable signal, confirming the specific detection of the sequence *blaCTX-M-15*. The phase variation for the *blaCTX-M-15* synthetic DNA target (at 10 nM) was 0.08 × 2πrad, while for the synthetic negative control (at 10 nM) was almost negligible, 1.3 × 10^−3^ × 2πrad. Figure 3C shows the average signal obtained for the specific *blaCTX-M-15* synthetic DNA target and for the negative controls.

### 3.2. Evaluation of the Genes Encoding Betalactamase CTX-M-15 and Carbapenemase NDM-5

#### 3.2.1. Betalactamase *CTX-M-15*

**Figure 4:**
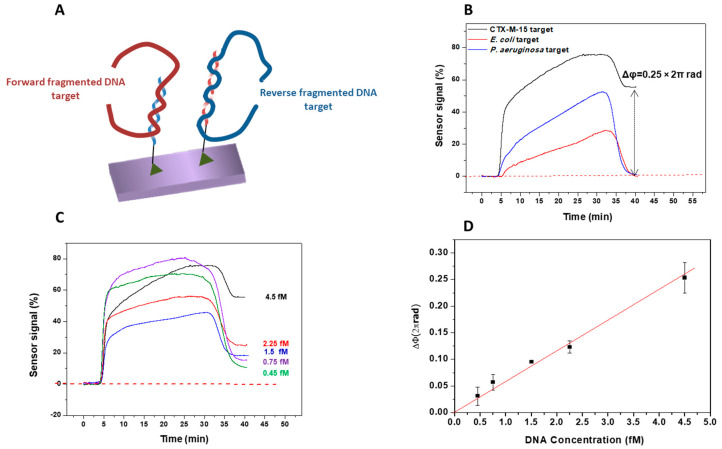
Detection of *blaCTX-M-15* gene by the BiMW biosensor. (**A**) Schematic representation to detect both DNA strands using forward and reverse probes. (**B**) Real-time detection of *blaCTX-M-15* gene at 4.5 fM (black line), non-specific *Pseudomonas aeruginosa* at 3.4 fM (blue line), and *Escherichia coli* without *blaCTX-M-15* gene at 2.9 fM (red line). (**C**) Real-time interferometric signals of *blaCTX-M-15* at several concentrations (0.45–4.5 fM). (**D**) Calibration curve obtained for the range of concentrations analyzed. The curve was fitted to a linear regression (y = a + bx, R^2^ = 0.994). All the measurements were performed in triplicate, and the data are shown as the mean + SD.

In order to demonstrate the biosensor capability for the analysis of real samples, the next step was the evaluation of real bacterial culture samples. Two sequences (forward and reverse), specific for the *blaCTX-M-15* gene, were selected as probes and immobilized on the sensor surface (see Figure 4A). The use of forward and reverse probes was intended for increasing the possibility to detect both DNA strands, and to increase the sensitivity of the biosensor.

After the preparation steps, the samples of the *blaCTX-M-15* gene, with concentrations ranging from 0.45 to 4.5 fM, were sequentially flowed over the biosensor surface. Figure 4B shows the evaluation of the specificity of the *blaCTX-M-15* probes in the BiMW biosensor by monitoring the sensor signal after injection of different negative controls. We tested non-specific DNA (< 1000 bp) from P. aeruginosa at a concentration of 3.4 fM and from E. coli lacking *blaCTX-M-15* gene at a concentration of 2.9 fM. These concentrations of the negative controls are similar to the highest concentration of the *blaCTX-M-15* target used. These signals show a phase variation of 0.25 × 2πrad for the detection of 4.5 fM *blaCTX-M-15* gene, and negative control samples did not give a significant signal, confirming that the response contribution comes exclusively from the *blaCTX-M-15* target. In addition, the biosensor could detect in real time the *blaCTX-M-15* gene interactions at a concentration as low as 0.45 fM, directly and without pre- or post-amplification steps (see Figure 4C). From the signals obtained for each concentration, a calibration curve was generated. The limit of detection, calculated from the linear fitting of the representation of the phase variation vs. DNA concentrations, and considering the minimum measurable signal set as three times the standard deviation of the baseline (8.5 × 10^−5^ × 2πrad), yielded a value of only 30 aM (~10^5^ CFU mL^−1^), and the limit of the quantification (LOQ) was 250 aM. The data showed a good coefficient of determination (R^2^ = 0.994), which is considered as a very encouraging result taking into account the high complexity of the sample tested (see Figure 4D). These results confirmed that the methodology was highly sensitive and specific for detecting fragmented DNA targets.

#### 3.2.2. Carbapenemase *NDM-5*

**Figure 5:**
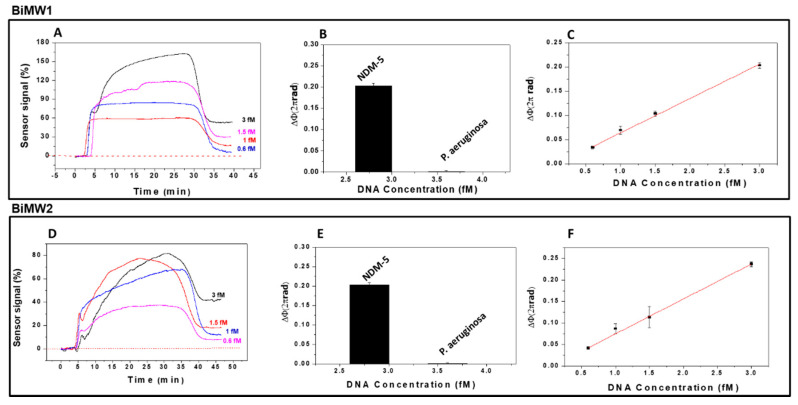
Detection of *blaNDM-5* gene with two BiMW biosensors (BiMW1 and BiMW2). (**A**,**D**) Real-time interferometric signals for different concentrations of *blaNDM-5* gene (0.6–3 fM) analyzed over a sensor chip with the *blaNDM-5* probe immobilized. (**B**,**E**) Comparison of triplicate measurements of *blaNDM-5* DNA target (3 fM) versus *P. aeruginosa* DNA (3.4 fM). (**C**,**F**) Calibration curves obtained for the range of concentrations analyzed. All the measurements were performed in triplicate, and the data are shown as mean + SD.

In order to detect the presence of the *blaNDM-5* gene in *E. coli* samples and compare it with the *blaCTX-M-15* gene, we employed a forward probe with a short nucleotide spacer. Moreover, to assess efficiency and reproducibility among BiMW sensor devices, the detection of the *blaNDM-5* gene was done using two different sensors (BiMW1 and BiMW2). The biosensor BiMW 1 was calibrated with genomic samples prepared following the same pre-treatment, and after thermal denaturation, genomic DNA was tested in triplicates at different concentrations (see Figure 5A). The lowest concentration employed (0.6 fM) produced a phase variation of 0.035 × 2πrad, while the highest produced a phase variation of 0.20 × 2πrad. These results demonstrate the capability of the BiMW biosensor to detect the *blaNDM-5* gene at very low concentration without the need for any signal amplification or previous PCR amplification.

Once we demonstrated the recognition of the *blaNDM-5* gene, we confirmed the specificity of the assay by evaluating a *P. aeruginosa* DNA as negative control (3.4 fM). As shown in Figure 5B, the designed probe showed a high specificity for the sample with the *blaNDM-5* gene from *E. coli*, and a negligible signal for the negative control. As previously observed with the *blaCTX-M-15* gene, the response compared with the negative control was significantly higher (0.21 × 2πrad for *blaNDM-5* gene and 0.004 × 2πrad for the negative control), which confirms the specificity of the probes. The resulting calibration curve is shown in Figure 5C, from which a limit of detection of 28 aM (~10^5^ CFU mL^−1^) and LOQ of 234 aM were calculated. The data showed a good coefficient of determination (R^2^ = 0.996). 

As Figure 5 shows, we also evaluated the reproducibility of the results of the evaluation of the *blaNDM-5* gene in another BiMW sensor device (BiMW2), obtaining results similar to those obtained with BiMW1 (Figure 5D) for the same evaluated concentrations. The specificity of the BiMW2 biosensor was also evaluated with a *Pseudomonas* DNA negative control (3.4 fM) (Figure 5E). The calibration curve showed a good linear fitting (R^2^ = 0.998), obtaining a limit of the detection of 24 aM and LOQ of 205 aM. As Figure 5F shows, these results and the real-time signals are comparable with the BiMW1 results (LOD: 28 aM). 

The BiMW biosensor for the *blaNDM-5* gene detection demonstrated high sensitivity, real-time monitoring, and reproducibility in two BiMW sensor devices employed for a range of very low concentrations, without the need for any amplification or labeling steps. The designed strategy can be further expanded to evaluate any other relevant resistance sequence genes present in other pathogens.

## 4. Conclusions

We have demonstrated the efficiency and ultra-sensitivity of a BiMW nanophotonic biosensor for the detection of multidrug resistant genes directly from bacterial cultures of *Escherichia coli*. Specifically, we implemented the biosensor for the detection of *blaCTX-M-15* and *blaNDM-5* antimicrobial-resistant gene sequences. This BiMW biosensor methodology is fast, ultra-sensitive (attomolar levels), reproducible, and has the potentially to be extended to a wide variety of genes from other pathogens. 

Direct bacterial genomic DNA detection is an attractive goal for diagnostics, allowing faster analytical responses and cost reduction of the assays, compared with current available methods such as PCR. Our strategy involved the immobilization of two probes (forward and reverse) to capture both DNA strands, as done in PCR. This methodology provides an extraordinary opportunity for molecular microbiology diagnosis, as this biosensor provides considerable advantages over PCR, particularly in terms of assay time and cost, and could be easily adapted for point-of-care applications. This BiMW biosensor, with average LOD in the range of 25 aM and LOQ of 205 aM, represents one of the most sensitive approaches for whole genomic DNA detection without amplification or labeling steps. In addition, the reproducibility was confirmed with two BiMW sensor devices, and discrimination between different genomic DNA from several bacteria was achieved. 

In view of the high sensitivity exhibited by the BiMW biosensor, it could potentially be used for the early detection of infections caused by multidrug resistant bacteria, contributing to the early implementation of accurate therapeutic interventions.

## Figures and Tables

**Table 1:** Probe and Target Sequences.

Probe	Target Sequences
**CTX-M-15 synthetic target**	5′ TTT GCG CAT ACA GCG GCA CAC TTC CTA ACA ACA GCG TGA CGG TTG CCG TCG CCA TCA GCG TGA ACT GGC GCA GTG ATT TTT TAA CCA T 3′
**Negative synthetic control**	5′ ACG CUG UCG GUG AGU 3′
**CTX-M-15 F probe**	5′ NH2-(CH2)6-TTG TTA GGA AGT GTG CCG 3′
**CTX-M-15 R probe**	5′ NH2-(CH2)6-TAA GTG ACC AGA ATC AGC GG 3′
**NDM-5 probe**	5′ NH2-(CH2)6-TTT TTT TTT TGC CCA ATA TTA TGC ACC CG 3′
